# Dispersion of Graphene Oxide: Evaluating Ionic Surfactants for Nanocomposite Cement Applications

**DOI:** 10.3390/nano16100632

**Published:** 2026-05-19

**Authors:** Sadixa Baral, Ramesh Raghavendra, Ken Thomas, Raja Das

**Affiliations:** 1 Department of Engineering Technology, South East Technological University, X91 K0EK Waterford, Ireland; sadixa.baral@postgrad.wit.ie (S.B.); ramesh.raghavendra@setu.ie (R.R.); ken.thomas@setu.ie (K.T.); 2 School of Engineering and Built Environment, South East Technological University, X91 K0EK Waterford, Ireland; 3 South East Applied Materials (SEAM) Research Centre, South East Technological University, X91 TX03 Waterford, Ireland

**Keywords:** graphene oxide, nanomaterials, dispersion, calcium hydroxide, cementitious composite, ionic surfactant, colloidal stability, Ca^2+^ induced aggregation, SDBS, CTAB

## Abstract

Graphene oxide (GO) has been widely investigated as a nanoreinforcement for cementitious composites; however, its effectiveness depends on stable dispersion within the highly alkaline, calcium-rich environment of fresh cement paste. This study evaluates the dispersion behaviour of GO in deionised (DI) water and saturated calcium hydroxide (Ca(OH)_2_) under controlled conditions and assesses the effectiveness of anionic and cationic surfactants in both environments. GO was synthesised using the modified Hummers method and verified by comprehensive physicochemical characterisation. Dispersion stability was assessed using UV-Vis spectroscopy at GO concentrations of 0.04–0.08 mg/mL in DI water, and the 0.08 mg/mL system was further studied in saturated Ca(OH)_2_ with and without sodium dodecylbenzene sulphonate (SDBS) and cetyltrimethylammonium bromide (CTAB) at a 1:1 mass ratio. Zeta potential and dynamic light scattering measurements were performed to understand the relation between the surface charge and agglomeration of GO. In DI water, GO retained close to 70% of its initial absorbance after 60 min, and both surfactants improved retention to above 90%. In saturated Ca(OH)_2_, retention fell to approximately 40%, and neither surfactant restored stability despite producing zeta values that would conventionally support stable dispersion. The findings indicate that GO aggregation in calcium ion (Ca^2+^)-rich alkaline environments is not governed by net surface charge alone, consistent with the established mechanism of Ca^2+^ chemical cross-linking with GO carboxyl groups.

## 1. Introduction

Cementitious composites are among the most widely produced and consumed construction materials due to the availability of raw materials, well-established production processes, relatively low cost, and the ease of application in civil infrastructure. They are valued for their mechanical robustness, versatility, and particularly their high compressive strength [1]. However, conventional Portland cement-based composites are fundamentally limited due to multiple weaknesses. Cement production is responsible for over 8% of global carbon emissions [2,3]. This environmental burden has intensified the search for low-carbon, high-performance cementitious systems, in which partial replacements of cement with nanomaterial-modified admixtures have emerged as a promising route towards carbon neutrality in construction [4]. They have a brittle nature, low tensile strength, and poor resistance to crack initiation and propagation. These intrinsic shortcomings lead to the formation and growth of microcracks, which can compromise their long-term structural performance [5,6]. Consequently, improving the tensile behaviour, crack resistance, and overall performance of cementitious composites has become a critical focus of research, motivating the development of advanced reinforcement strategies. These include the incorporation of nanomaterials to enhance microstructural performance and overall mechanical properties [7]. Over the past two decades, the incorporation of nanomaterials into cementitious matrices has become an important area of research in construction materials. Nanoscale additives such as nano-silica [8,9,10], nano-alumina [11], and carbon nanotubes/nanofibers (CNTs/CNFs) [12,13,14] have shown significant potential in improving the mechanical properties, microstructure, and durability of cement composites. Among these nanomaterials, carbon-based nanomaterials have attracted particular attention. In this context, graphene [15,16,17,18,19,20,21,22,23,24,25] and its nanosheets have emerged as a promising material for cement-based reinforcement. However, the direct dispersion of graphene in the cement matrix remains challenging, which has limited widespread application [25].

Researchers have increasingly focused on graphene oxide (GO) [19,26,27,28,29], a derivative of graphene. GO is a two-dimensional nanomaterial derived from chemical oxidation and exfoliation of graphite [30]. Unlike pristine graphene, which is chemically inert and strongly hydrophobic, GO carries abundant oxygen-containing functional groups, i.e., hydroxyl (-OH), epoxy (-CH(O)CH-), carboxyl (-COOH), and carbonyl (C=O), which are distributed across its basal planes and edges [27,31]. These functional groups reduce the intermolecular attraction and increase electrostatic repulsion in aqueous solutions, thereby improving the dispersibility of GO [32,33]. Furthermore, the presence of these oxygen-containing functional groups provides active sites for chemical interactions and further functionalisation, which enhances interfacial bonding with the cement matrix [34]. Owing to these characteristics, GO has been extensively investigated as a nanoreinforcement for cementitious applications.

Presently, the substantial literature on GO-reinforced cement composites reveals several consistent and well-produced trends. A foundational study has shown that, at a dosage of just 0.05 wt% by mass of cement, GO has been shown to increase compressive strength by 15–33% and flexural strength by 41–59% [26]. These improvements were attributed to the crack-bridging capacity of GO’s two-dimensional sheet geometry and enhanced mechanical interlocking between the GO morphology and the cement matrix [26]. Comparable gains have been reported at even lower dosages. At an optimised content of 0.03 wt%, GO sheets were found to regulate the formation of flower-like hydration crystals through the template effect, increasing the tensile, flexural, and compressive strengths by 78.6%, 60.7%, and 38.9%, respectively [33]. The improvements reduce brittleness and increase composite toughness. These mechanical improvements are generally attributed to several reinforcing mechanisms. The large surface area of GO provides nucleation sites that promote the formation of calcium silicate hydrate (C-S-H) gel during cement hydration [33]. In addition, its oxygen-containing functional groups enable chemical interactions with hydration products at the nanoscale. Recent mechanistic investigations of carbon nanotube–cement systems have further shown that nanocarbons influence hydration not primarily through nucleation but by modulating Ca^2+^ distribution, refining C-S-H gel structure, and promoting the formation of high-density C-S-H, which collectively enhance the elastic and viscoelastic behaviour of the hardened matrix [35]. These findings underline that the interaction between nanocarbons and the Ca^2+^-rich hydration environment is central to their reinforcing action. On the other hand, its sheet geometry bridges microcracks and controls their propagation from the nanoscale upward [26,33].

A recurring theme in the GO–cement literature, and arguably one of the most significant practical challenges in this field, is the strong dependence of GO’s reinforcing effectiveness on its dispersion within the cement matrix. Comprehensive reviews of graphene-modified cementitious composites have constantly identified dispersion as the central bottleneck restricting the translation of laboratory-scale mechanical gains into large-scale engineering applications [36]. The mechanical and microstructural enhancements attributed to GO rely on its uniform distribution throughout the composite. Without uniform distribution, the reinforcing mechanisms cannot be realised [37]. Poorly dispersed GO also limits the practical utilisation of its properties and restricts its wider application in cement-based materials [38]. This challenge is fundamentally rooted in the colloidal behaviour of GO under the ionic conditions of fresh cement mix, a problem that deionised water-based dispersion protocols cannot adequately replicate or predict.

Fresh cement paste represents a strong alkaline and Ca^2+^-rich environment, to which GO is particularly sensitive. Previous studies have shown that the destabilisation of GO in cementitious systems is governed primarily by the interaction of Ca^2+^ with oxygen-containing functional groups on GO sheets, rather than the high alkalinity alone [39]. In particular, Ca^2+^ can form complexes with carboxylate groups (-COO^−^) located at the edges of GO sheets, acting as a bridging cross-linker between adjacent layers. This interaction reduces the electrostatic repulsion that normally maintains colloidal stability in deionised water, thereby promoting aggregation. Experimental evidence indicates that Ca^2+^ concentrations as low as 2.2 mM are sufficient for inducing rapid and visible flocculation of GO suspensions [39]. Rapid aggregation has also been reported when GO is exposed to Ca^2+^-containing alkaline solutions, such as saturated Ca(OH)_2_, which is commonly used as a simplified model of cement pore solutions in experimental studies [39]. As a result, a GO suspension that appears well dispersed in deionised water does not necessarily retain the same stability when introduced into the Ca^2+^-rich environment typical of cementitious systems [38].

Surfactant-assisted dispersion has been widely explored as a practical strategy to improve the stability of graphene-based nanomaterial suspensions [40,41,42,43,44]. Both anionic surfactants, such as sodium dodecylbenzene sulphonate (SDBS), and cationic surfactants, such as cetyltrimethylammonium bromide (CTAB), have been applied to graphene- and carbon-related nanomaterial dispersions to enhance colloidal stability through electrostatic interactions with the sheet surface [40,43]. While these approaches have demonstrated improved dispersion in deionised water, their effectiveness in Ca^2+^-rich alkaline environments relevant to cementitious systems remains uncertain [40]. Recent advances have explored alternative routes to improve GO dispersion. For instance, concentrated water-stable graphene nanofluid admixtures have been developed to replace mixing water in concrete production directly. These systems achieve notable gains in compressive and flexural strength while reducing cement consumption and associated carbon [4]. Such developments demonstrate the practical value of pre-dispersed aqueous nanocarbon systems. However, they also highlight the need for a deeper understanding of the underlying colloidal behaviour that governs their stability. Previous studies have shown that physical dispersion strategies, including surfactant addition, can improve GO dispersibility in water but may not fully prevent reaggregation when exposed to Ca^2+^ [39,45]. However, despite these advances, the behaviour of conventional ionic surfactants in Ca^2+^-rich alkaline environments has not been systematically evaluated. In particular, direct comparisons of anionic and cationic surfactants such as SDBS and CTAB under saturated Ca(OH)_2_ conditions remain limited, motivating the present investigation.

Several previous studies have specifically addressed the aggregation and flocculation of GO in cement-based and cement-simulating environments and collectively established that the highly alkaline, Ca^2+^-rich pore solution causes severe destabilisation of GO dispersion. Investigations of GO dispersion in water, alkali, and a range of ionic species using UV-Vis spectroscopy have identified high alkalinity and Ca^2+^ as the key factors inducing GO agglomeration in a simulated cement pore solution. The polycarboxylate-based superplasticisers are reported as the most promising among the commercial admixtures tested for dispersing GO in an alkaline cement environment [45]. Mechanistic work comparing GO behaviour in Ca(OH)_2_, CaCl_2_, and NaOH solutions has further shown that Ca^2+^ complexation with functional groups, rather than alkalinity, is the primary factor responsible for the immediate aggregation of GO; the high pH of Ca(OH)_2_ solutions additionally deprotonates the carboxyl group on GO, providing more carboxylate (COO^−^) groups available for Ca^2+^ cross-linking [39]. Consistent with this, marked flocculation of GO has been reported to occur even at very low Ca(OH)_2_ concentrations, with both the -COOH group and the long sidechain of polycarboxylate-based superplasticisers identified as more effective than -OH groups at delaying flocculation [46]. These studies collectively establish Ca^2+^-mediated cross-linking of GO carboxyl groups as the dominant destabilising mechanism in the cementitious pore solution. However, a direct controlled comparison between anionic and cationic surfactants under identical dosage and conditions in both DI water and saturated Ca(OH)_2_ has not been systematically reported.

The present study addresses this gap directly by systematically evaluating the dispersion behaviour of GO under conditions relevant to cementitious environments. GO was synthesised via the modified Hummers method and characterised using scanning electron microscopy (SEM), Attenuated Total Reflectance-Fourier Transform Infrared spectroscopy (ATR-FTIR), Raman spectroscopy, and X-ray diffraction (XRD). Dispersion stability was then assessed in DI water, in saturated Ca(OH)_2_, and in the presence of ionic surfactants SDBS and CTAB using UV-Vis sedimentation measurements supported by zeta potential and dynamic light scattering analyses to determine the electrostatic state and aggregate size of each system. The results provide insight into the effectiveness and limitations of simple ionic surfactants under Ca^2+^-rich alkaline conditions, contributing to the development of strategies for reliable GO incorporation into cement-based nanocomposites.

The scope of the present work is intentionally restricted to establishing a mechanistically grounded baseline for GO dispersion behaviour under simplified and controlled conditions. A fixed GO-to-surfactant mass ratio of 1:1 and a standardised sonication protocol (130 W, 60% amplitude, 15 min) were applied across all systems to isolate the influence of two variables of primary interest, namely the dispersion medium (DI water and saturated Ca(OH)_2_ solution) and the surfactant charge type (anionic SDBS versus cationic CTAB). Keeping the dosage and energy input constant allows a direct comparison of the effects of the dispersion medium and surfactant type on colloidal stability by minimising interfering variables. This baseline approach is the necessary first step before more complex variables can be meaningfully examined. Aspects such as dosage-dependent behaviour, particle-size kinetics, mixed surfactant systems, steric stabilisation, comparison with commercial cement superplasticisers, and incorporation into cement systems are beyond the scope of this study and will be addressed in subsequent works, for which the present study provides a foundational dispersion baseline.

## 2. Methodology

### 2.1. Materials and Reagents

In this study, graphite flakes (median particle size 7–8 µm, 99% purity) were procured from Thermo Scientific (Ward Hill, MA, USA). Sodium nitrate (NaNO_3_) was obtained from Sigma Aldrich (UK). Potassium permanganate (KMnO_4_, 98%, extra pure), hydrogen peroxide (H_2_O_2_, 30%, laboratory reagent grade), hydrochloric acid (HCl, specific gravity 1.18, ~37%, analytical reagent grade) (UK), and sulfuric acid (H_2_SO_4_, specific gravity 1.83, ≥95%, analytical reagent grade) were obtained from Fisher Scientific (UK). Hexadecyltrimethylammonium bromide (CTAB—98%) was procured from Thermo Scientific (UK), and sodium dodecylbenzene sulfonat (SDBS) was from MP Biomedicals (Solon OH, USA), and they were used as dispersion additives. Calcium hydroxide (Ca(OH)_2_) was obtained from Thermo Scientific (UK). All chemicals were used without further purification. DI water was used for all dispersion preparation.

### 2.2. Experimental Design

The experimental program was designed to systematically investigate the dispersion behaviour of GO under an aqueous environment relevant to cementitious systems. This study comprised the three main stages: (i) synthesis of GO using the modified Hummers method, (ii) physicochemical characterisation of the synthesised GO, and (iii) evaluation of GO dispersion stability in varying ionic conditions using UV-Vis spectroscopy.

Baseline dispersion was first established by dispersing GO in DI water at three different concentrations (0.04, 0.06, and 0.08 mg/mL). These suspensions were subjected to an identical sonication protocol (15 min) to assess the influence of GO concentration on dispersion behaviour in a low-ionic-strength medium. The highest GO concentration, i.e., 0.08 mg/mL, was selected for further investigation to represent a more challenging dispersion condition.

Following the baseline study, dispersion behaviour at the selected GO concentration was examined under various chemical environments. Firstly, 0.08 mg/mL GO was dispersed in a saturated Ca(OH)_2_ solution. Here, saturated Ca(OH)_2_ was used as a simplified model system to represent the highly alkaline and calcium-rich conditions typical of cement pore solutions. The effect of additives was then evaluated by introducing an anionic surfactant, SDBS, and a cationic surfactant, CTAB, first in water and then in saturated Ca(OH)_2_. This stepwise approach enabled a comparative assessment of the dispersion behaviour of GO across progressively more chemically aggressive environments.

### 2.3. Synthesis of GO

The commonly used method for GO synthesis is Hummers’ method [47]. In this method of synthesis, GO was synthesised by adopting a modified Hummers’ method. To begin with, 2 g of graphite flakes and 1 g of NaNO_3_ were dispersed in 50 mL of concentrated H_2_SO_4_ under continuous stirring in an ice bath (<5 °C). The mixture was placed in an ice bath to control the exothermic heat generated. Then, 6 g of KMnO_4_ was slowly added while maintaining the reaction temperature below 20 °C, followed by oxidation at 35 °C on a hot plate for 16 h. The beaker was then plunged into an ice bath, and the reaction was quenched by adding 500 mL of DI water. Then, 8 mL of H_2_O_2_ was added to terminate the reaction and to eliminate excess KMnO_4_. The resulting suspension was allowed to settle for 12 h, centrifuged, and repeatedly washed with 10% HCl and deionised water until a neutral pH was achieved. The final GO product was vacuum-dried at 40 °C for 72 h to obtain the GO film.

### 2.4. Characterisation Methods

To confirm the successful synthesis of GO, a range of structural and spectroscopic characterisation techniques was employed. XRD measurements were carried out using a Rigaku MiniFlex diffractometer (Tokyo, Japan) with Cu Kα radiation. The surface morphology of the samples was analysed by SEM on a HITACHI (Tokyo, Japan) instrument. The functional group analysis was performed using ATR-FTIR spectroscopy via a Bruker LUMOS II system (Massachusetts, USA), and Raman spectroscopy was carried out using a Thermo Scientific DXR3 Raman microscope (Massachusetts, USA), equipped with a 785 nm excitation laser.

### 2.5. Preparation of GO Dispersion

GO dispersions were prepared using DI water as the primary dispersion medium. In order to establish baseline dispersion behaviour, GO was dispersed at concentrations of 0.04, 0.06, and 0.08 mg/mL. The required mass of GO was added to DI water and subjected to probe ultrasonication using a Sonics & Materials, Inc. VCX 130PB sonicator (Newtown, CT, USA) (130 W, 20 kHz) at an amplitude of 60% for a total duration of 15 min. The sonication time of 15 min was selected based on the literature [40]. It was reported that the dispersion degree of aqueous graphene–SDBS suspensions measured by UV-Vis absorbance reaches a plateau at approximately 15 min of sonication, beyond which no further improvement in dispersion is observed [40]. Sonication was performed in intermittent cycles of 2 min 30 s followed by a 10 s pause to minimise overheating. Samples were maintained in an ice bath to limit the temperature rise during sonication.

The temporal stability of the baseline dispersion was evaluated using a Shimadzu UV dual beam spectrophotometer (Kyoto, Japan) by recording absorbance immediately after sonication and at regular intervals up to 60 min (0 min, 10 min, 20 min, 30 min, 40 min, 50 min, and 60 min). Parallelly, visual assessment of dispersion stability was conducted with images of dispersion vials captured at selected time points (0 min, 30 min, and 60 min).

GO dispersion of 0.08 mg/mL was prepared with a saturated Ca(OH)_2_ solution to simulate the high-alkaline, calcium-rich environment characteristic of cement pore solution. The suspension was subjected to the same sonication protocol as described above. Dispersion stability was evaluated using UV-Vis spectroscopy at a fixed wavelength of 230 nm, corresponding to the characteristic π-π* transition of GO, which provides high sensitivity for monitoring dispersion stability [45,48]. Visual observations were also recorded to observe the sedimentation behaviour.

The influence of ionic additives on GO dispersion was examined using two additives, SDBS and CTAB. For these experiments, a 0.08 mg/mL GO dispersion in DI water was prepared with the addition of surfactant at a GO-to-additive mass ratio of 1:1. Following sonication under identical conditions, dispersion stability was evaluated through time-dependent UV-Vis absorbance measurements at 230 nm.

To assess the combined effect of the alkaline environment and ionic additives, GO dispersions containing either SDBS or CTAB were also prepared in a Ca(OH)_2_ solution. These suspensions were subjected to the same preparation, sonication, and analytical procedures to enable direct comparisons across all dispersion conditions. Full UV-Vis spectra and raw absorbance data are provided in Supplementary Materials (Figures S2 and S3 and Tables S1 and S2). UV-Vis absorbance measurements reported in this study correspond to single representative runs per condition and are therefore presented without error bars. Although the experiments were carried out multiple times, consistent absorption behaviour was observed. Hence, the data are interpreted in terms of qualitative trends.

### 2.6. Zeta Potential and Particle Size Measurements

Zeta potential and hydrodynamic particle size measurements were performed using a Malvern Zetasizer Advance Ultra (Malvern Panalytical, Malvern, UK) equipped with disposable folded capillary zeta cells (DTS1070, Malvern Panalytical). Samples were prepared following the same protocol described in Section 2.5 at a GO concentration of 0.08 mg/mL and a GO-to-surfactant mass ratio of 1:1, where applicable. To ensure consistency across all systems, all measurements were carried out within 10 min of the end of sonication. The pH of each suspension was recorded immediately before measurement. Measurements were performed at 25 °C, with a 60 s equilibration time before each measurement. The results are reported as mean ± standard deviation of the three runs.

## 3. Results

### 3.1. GO Characterisation

#### 3.1.1. Raman Spectroscopy

**Figure 1:**
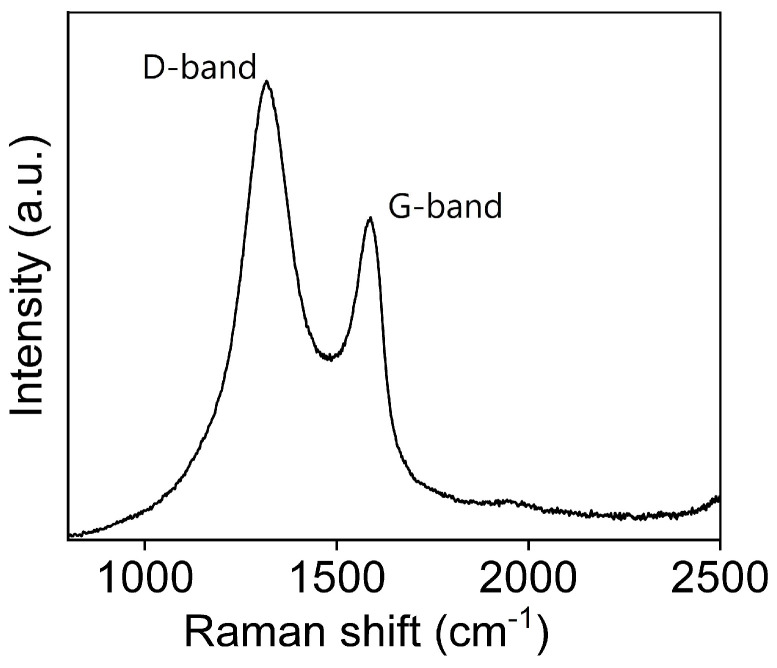
Raman spectrum of GO showing the presence of graphitic bands (D band at 1583 cm^−1^ and 1317 cm^−1^).

Raman spectroscopy is a widely used non-destructive technique for obtaining structural information of a carbon-based material [49]. The most prominent feature of the Raman spectrum of carbon-based material is the G and D peaks and their overtones in the observed spectra. The Raman spectrum of the synthesised GO is shown in Figure 1, which shows the presence of the graphitic band, G-band, and the defect band, D-band. We can observe the G-band at 1583 cm^−1^ and the D-band at 1317 cm^−1^. The G band occurs due to the band stretching of all sp^2^ bands in both types of rings and chains. This is due to in-plane optical vibrations. The D band arises from a defect-induced vibrational mode of sp^2^-bonded carbon rings and is linked to the presence of structural imperfections or disorder within the material [50]. The intensity ratio ($\frac{I_{D}}{I_{G}}$) of 1.43 indicates a high defect density, confirming the oxidation of graphite to GO.

#### 3.1.2. X-Ray Diffraction (XRD)

**Figure 2:**
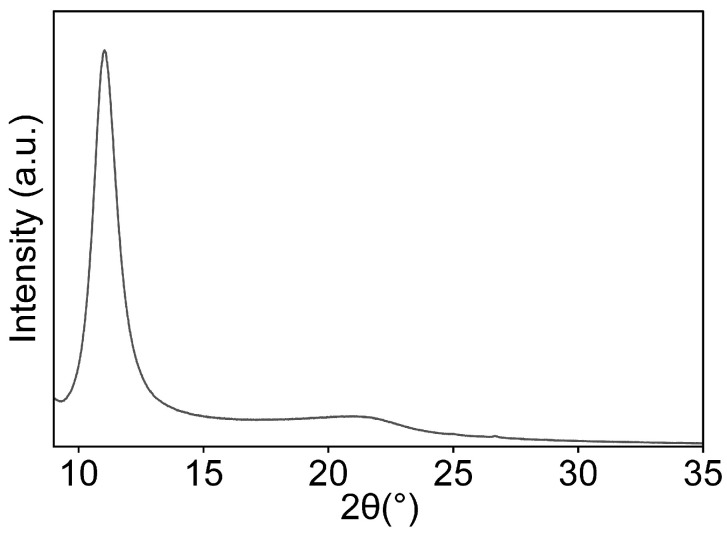
XRD spectrum of GO shows the diffraction peak at 2θ = 11°.

XRD is a commonly used method for characterising carbon-based materials, as it allows the determination of the average interlayer spacing between graphene planes and offers insight into the crystallographic orientation of individual carbon layers. The XRD pattern of GO is shown in Figure 2.

The figure shows a diffraction peak at 2θ = 11°, which is due to the oxidation of graphite. It is important to note that pristine graphite typically exhibits a diffraction peak near 26°. The loss of this peak and the emergence of a new peak around 11° indicate that the graphite flakes have been fully oxidised. This shift is attributed to successful oxidation, which introduces oxygen-containing functional groups between the graphite layers [2,51].

#### 3.1.3. Attenuated Total Reflectance Fourier Transform Infrared Spectroscopy (ATR-FTIR)

**Figure 3:**
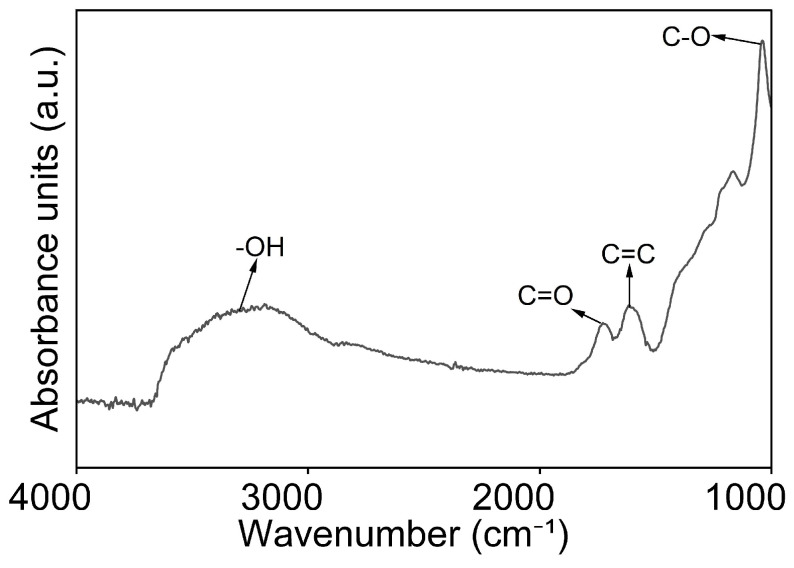
ATR-FTIR spectrum of GO shows characteristic peaks of hydroxyl at 3346 cm^−1^ (O-H stretching), carboxyl at 1717 cm^−1^ (C=O stretching), epoxide at 1044 cm^−1^ (C-O stretching), and C=C skeletal vibrations of the remaining sp^2^ carbon domains at 1592 cm^−1^.

In ATR-FTIR spectroscopy, absorbance peaks arise from the vibrational modes of the chemical bond, offering insights into the functional groups present in the sample. Figure 3 shows the ATR-FTIR spectrum of GO. Here, the spectrum shows characteristic peaks at 3346 cm^−1^ of hydroxyl (O-H stretching), 1717 cm^−1^ of carboxyl (C=O stretching), and 1044 cm^−1^ of epoxide (C-O stretching). The peak of 1592 cm^−1^ corresponds to C=C skeletal vibrations of the remaining sp^2^ carbon domains. The presence of oxygen-containing functional groups in the ATR-FTIR spectrum confirms that graphite has undergone oxidation [2]. Specifically, the detection of hydroxyl groups indicates the formation of hydrogen bonds with water molecules, which explains the hydrophilic behaviour of GO.

#### 3.1.4. Scanning Electron Microscopy (SEM)

**Figure 4:**
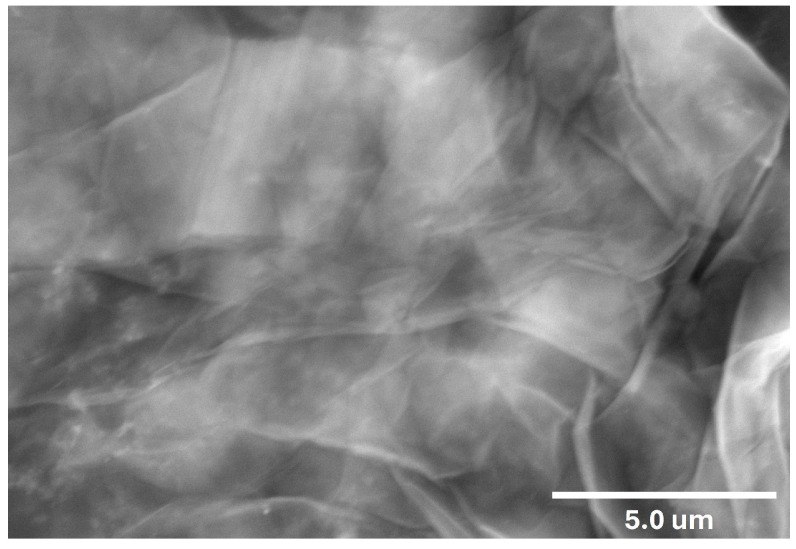
SEM of GO shows a wrinkled sheet-like morphology.

SEM is used to study the surface morphology of materials by scanning them with a focused electron beam. The SEM image of GO shown in Figure 4 displays wrinkled, sheet-like structures, which are typical of exfoliated GO. These wrinkles arise due to the presence of oxygen-containing functional groups that disturb the flat carbon layers, indicating the successful oxidation of graphite into GO [52]. More SEM micrographs of the synthesised GO are provided in Supplementary Materials (Figure S1).

### 3.2. Baseline Dispersion of GO

The baseline dispersion behaviour of GO was investigated at concentrations of 0.04, 0.06, and 0.08 mg/mL in DI water by monitoring the UV-Vis absorbance at 230 nm over time following sonication. Absorbance values were normalised with respect to the measurement at t = 0 min to allow direct comparisons between concentrations and to isolate the effect of concentration on dispersion stability, independent of the absolute absorbance magnitude.

**Figure 5:**
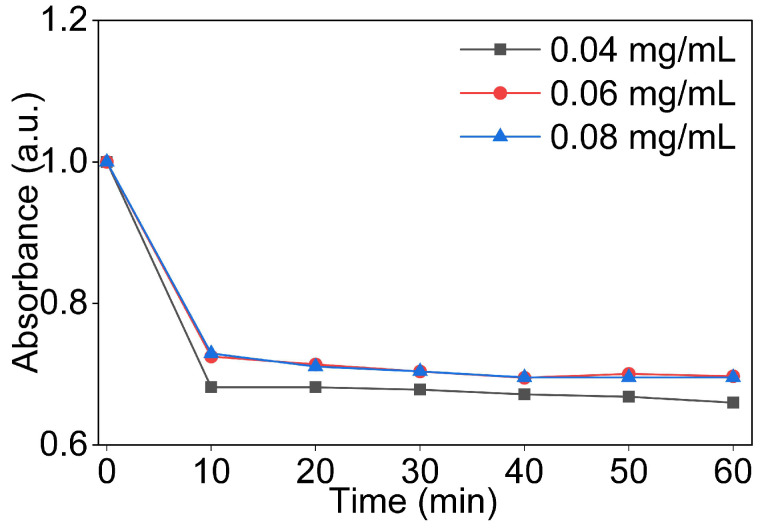
Normalised UV-Vis absorbance of GO dispersions in DI water as a function of time.

As shown in Figure 5, all three concentrations showed a characteristic two-phase absorbance profile. A sharp decrease in absorbance occurred within the first 10 min following sonication. After this, the absorbance stabilised and remained largely stable for the remainder of the 60 min observation period. The initial rapid decline is attributed to the sedimentation of larger GO aggregates and incompletely exfoliated particles that remain in the suspension immediately after sonication but are not stable in the colloidal state [53].

Across the three concentrations, absorbance dropped by 27–32% between t = 0 and t = 10 min, with the 0.04 mg/mL dispersion showing the largest decline. After this initial phase, absorbance changed only slightly over the remaining 50 min, by a few percent in all three dispersions. At t = 60 min, the 0.04 mg/mL dispersion retained approximately two-thirds of its initial absorbance, while 0.06 and 0.08 mf/mL dispersions both retained close to 70%. The comparable retention at the two higher concentrations indicates that, within the range tested, increasing GO concentrations from 0.06 to 0.08 mg/mL did not reduce dispersion stability. The 0.08 mg/mL dispersion also showed a stable plateau between t = 40 min and t = 60 min, with no measurable change in absorbance across these time points. This indicates that the remaining suspension was in a stable colloidal state.

**Figure 6:**
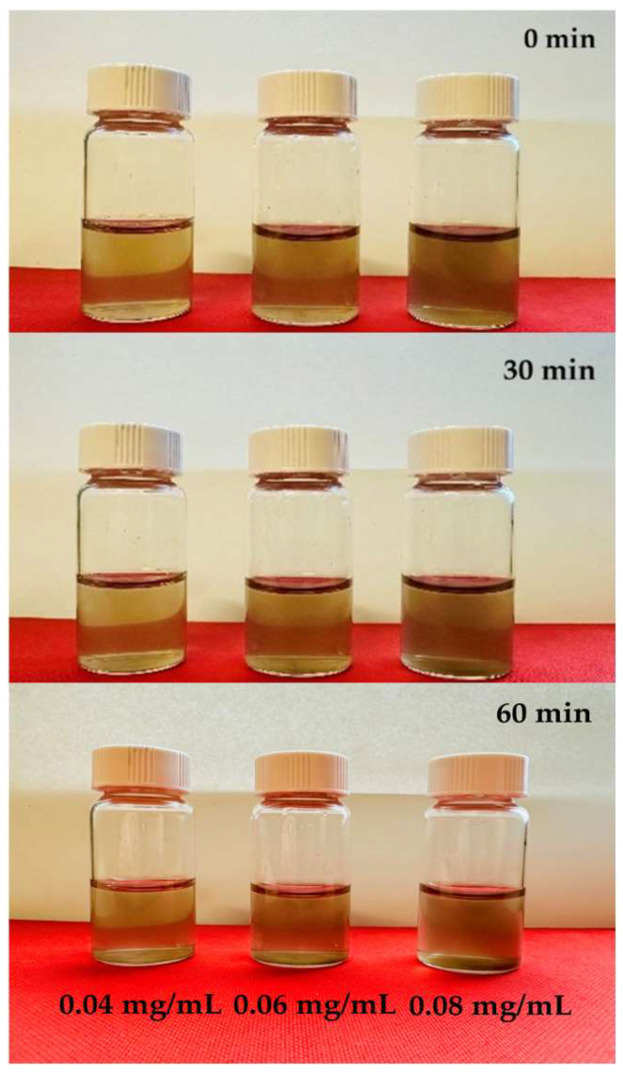
Visual inspection of GO dispersion stability in DI water with different concentrations at 0 min, 30 min, and 60 min.

Visual observation of the dispersion vials shown in Figure 6 was consistent with the spectroscopic data. No visible sedimentation was observed in any vial at t = 0 min or t = 30 min. At t = 60 min, faint settling was visible in the 0.04 mg/mL vial, while the 0.06 and 0.08 mg/mL vials remained visually homogeneous. This observation supports the UV-Vis results and highlights the greater sensitivity of spectroscopic measurement in detecting early-stage changes in dispersion uniformity that are not visible to the naked eye [54].

Based on these results, a GO concentration of 0.08 mg/mL was selected for all subsequent dispersion studies. This concentration provides the highest GO content in the tested range and is most relevant to the practical cement incorporation.

### 3.3. Dispersion in Saturated Ca(OH)_2_

A cement pore solution is highly alkaline, with pH values typically ranging from 12.5 to 13.5, and it contains elevated concentrations of Ca^2+^ [55]. To assess GO dispersions under conditions representative of this environment, 0.08 mg/mL GO dispersions were prepared in the saturated Ca(OH)_2_ solution, and their stability was monitored by UV-Vis spectroscopy at 230 nm over 60 min, following the same sonication protocol described in Section 2.5. This approach allowed assessment of GO colloidal stability under conditions similar to those encountered in fresh cement pore solution, which is critical for understanding its potential for incorporation into cement-based composites.

**Figure 7:**
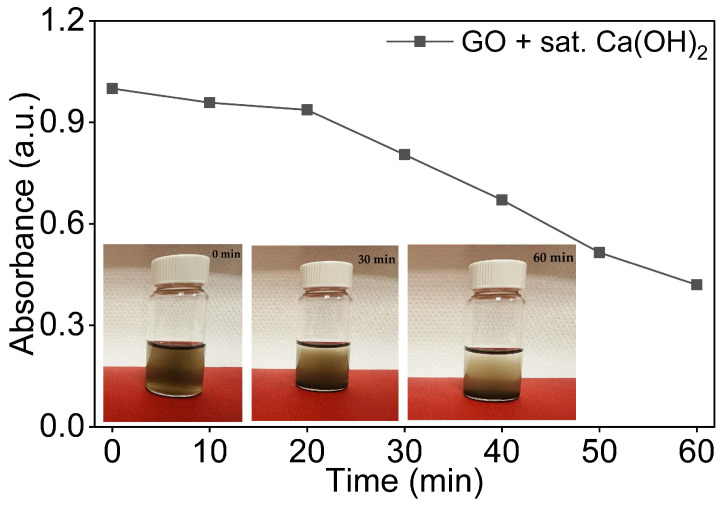
Normalised UV-Vis absorbance of 0.08 mg/mL GO dispersions in saturated Ca(OH)_2_ as a function of time with visual observation at 0 min, 30 min, and 60 min.

As shown in Figure 7, the GO/Ca(OH)_2_ dispersion exhibited a pronounced and continuous decline in absorbance, retaining approximately 40% of its initial value at t = 60 min compared to close to 70% for the equivalent DI water dispersions. The absorbance remained relatively stable during the first 20 min, with only a small decrease, before declining more rapidly between t = 20 min and t = 60 min. This behaviour is consistent with the Ca^2+^-induced charge screening and bridging flocculation, in which divalent Ca^2+^ coordinates with the carboxyl and epoxide groups on GO surfaces, neutralising the electrostatic repulsion and promoting progressive aggregation [56,57].

Visual inspection of the GO dispersion vial supported the UV-Vis observation, as shown in Figure 7. The dispersion appeared homogeneous immediately after sonication, with visible sedimentation evident by t = 30 min and substantial sediment accumulation at t = 60 min, consistent with the accelerated UV-Vis decay observed over the same period.

These results confirm that the Ca^2+^-rich alkaline environment characteristic of cement pore solutions severely compromises GO colloidal stability and highlight the need for a suitable stabilisation strategy before incorporation into cementitious composites, as explored in Section 3.4.

### 3.4. Effects of Ionic Additives

The colloidal stability of GO in aqueous media is governed primarily by electrostatic repulsion between the negatively charged GO sheets arising from ionised hydroxyl and carboxyl functional groups introduced during the oxidation process [56]. However, as demonstrated in Section 3.3, this inherent charge is insufficient to maintain dispersion stability in the ionic environment of the cement matrix. Ionic surfactants offer a route to enhance GO stability through additional electrostatic or steric mechanisms depending on their charge and concentration [37,40,42,45,58]. To investigate this, GO dispersions (0.08 mg/mL) were prepared with either the anionic surfactant SDBS or cationic surfactant CTAB at a 1:1 GO:surfactant mass ratio. Dispersion stability was monitored by UV-Vis spectroscopy at 230 nm over 60 min, following the same sonication protocol as previously discussed.

#### 3.4.1. GO Dispersions with SDBS in Water

SDBS is an anionic surfactant for which its aromatic hydrophobic tail adsorbs onto the basal plane of GO via hydrophobic and π–π stacking interactions, while its sulphonate head group projects into the solution, increasing the net negative charge of GO sheets and enhancing the electrostatic repulsion between them [42,58].

**Figure 8:**
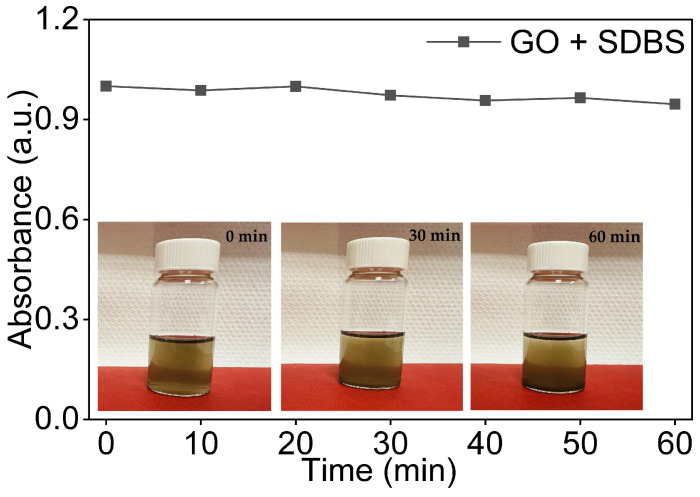
Normalised UV-Vis absorbance of 0.08 mg/mL GO dispersions in DI water and SDBS as a function of time with visual observation at 0 min, 30 min, and 60 min.

As shown in Figure 8, the GO/SDBS dispersion showed markedly improved stability compared to the baseline GO dispersion in DI water. The absorbance declined only slightly over the full 60 min observation period, retaining over 90% of its initial value at t = 60 min. This sharp initial drop of approximately 27% observed in the baseline dispersion (Section 3.2) between t = 0 and t = 10 min was largely suppressed in the presence of SDBS. From 10 min onward, the absorbance remained essentially constant, with no systematic decline, indicating that the suspended GO fraction had reached a stable colloidal state. Overall, retention at t = 60 min was substantially higher with SDBS than in the surfactant-free baseline (above 90% compared to close to 70%).

Visual inspection of the vials at 0, 30, and 60 min, as shown in Figure 8, supported the spectroscopic data. The dispersions remained visually homogeneous throughout, with no visible sedimentation observed at any time point, consistent with the minimal absorbance loss recorded by the UV-Vis.

These results confirm that SDBS effectively stabilises GO in aqueous dispersions. This is attributable to the dual role of the surfactant: adsorption onto the GO surfaces prevents sheet restacking, while the sulphonate head groups strengthen interparticle electrostatic repulsion [42].

#### 3.4.2. GO Dispersions with CTAB in Water

In contrast to SDBS, CTAB is a cationic surfactant for which its positively charged trimethylammonium head interacts with the negatively charged surface of GO through electrostatic attraction, while its hexadecyl tail adsorbs onto the GO basal plane via hydrophobic interaction [43]. Unlike SDBS, which reinforces the inherited negative charge of GO, CTAB partially neutralises it, which is a mechanistic distinction with direct consequences for colloidal stability.

**Figure 9:**
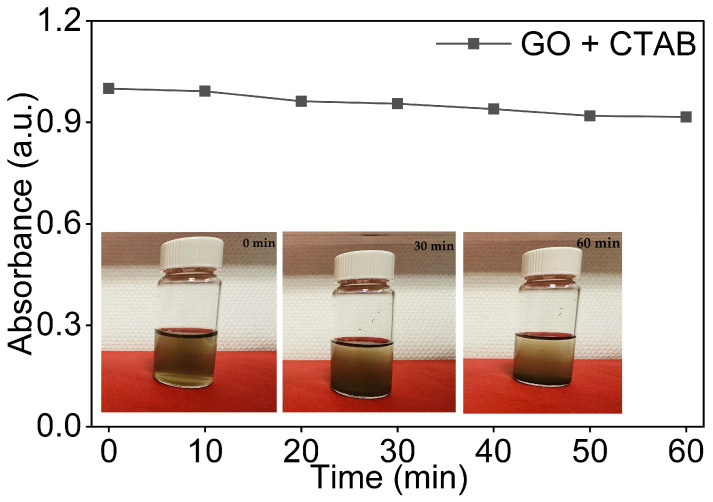
Normalised UV-Vis absorbance of 0.08 mg/mL GO dispersions in DI water and CTAB as a function of time with visual observation at 0 min, 30 min, and 60 min.

As shown in Figure 9, the GO/CTAB dispersion retained above 90% of its initial absorbance at t = 60 min, representing a substantial improvement over the baseline dispersion of the GO dispersion in DI water (close to 70%). However, in contrast to the GO/SDBS system, absorbance declined continuously and monotonically throughout the 60 min observation period, with no stabilisation plateau. This gradual and uninterrupted decay indicates that aggregation progressed steadily without the dispersion reaching a stable colloidal state within the observation window.

This behaviour is consistent with the partial charge neutralisation of GO surfaces by the adsorbed CTAB cations, which reduces the magnitude of electrostatic repulsion between sheets and allows slow, ongoing aggregation [43].

Visual inspection of vials taken at 0, 30, and 60 min in Figure 9 was consistent with the UV-Vis data. The dispersion appeared uniform immediately after sonication, with faint sedimentation visible by 60 min, consistent with the continuous absorbance decline recorded.

Overall, the GO/CTAB dispersion showed lower stability than the GO/SDBS system and did not reach a stable plateau within the observation period, although both surfactants markedly improved dispersion stability compared to the surfactant-free baseline in DI water.

### 3.5. Combined Effect of Additives in Saturated Ca(OH)_2_

As demonstrated in Section 3.3, the Ca^2+^-rich alkaline environment of the saturated Ca(OH)_2_ solution severely compromised GO colloidal stability, reducing absorbance retention at 60 min to approximately 40% compared to close to 70% in DI water. The destabilisation was attributed to Ca^2+^-mediated charge screening and bridging flocculation of GO sheets [56,59]. While Section 3.4.1 and Section 3.4.2 demonstrated that both SDBS and CTAB effectively enhanced GO stability in DI water, the performance of these surfactants in a calcium-rich alkaline medium directly relevant to the cement matrix remains to be established. To address this, GO dispersions containing either SDBS or CTAB were prepared in a saturated Ca(OH)_2_ solution, and their stability was evaluated using UV-Vis spectroscopy and visual inspection. The same sonication protocol as described in Section 2.5 was followed.

#### 3.5.1. GO Dispersions with SDBS in Sat. Ca(OH)_2_

**Figure 10:**
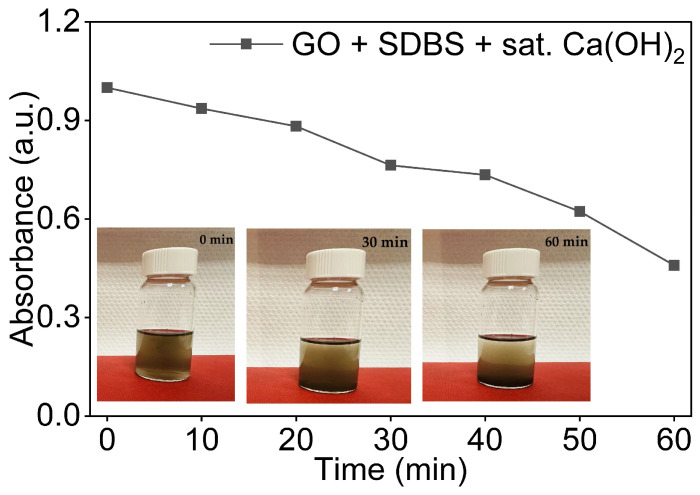
Normalised UV-Vis absorbance of 0.08 mg/mL GO dispersions in saturated Ca(OH)_2_ and SDBS as a function of time with visual observation at 0 min, 30 min, and 60 min.

The dispersion of GO in the saturated Ca(OH)_2_ in the presence of the anionic surfactant SDBS was evaluated using UV-Vis spectroscopy. A GO: SDBS ratio of 1:1 was used following the same sonication protocol. As shown in Figure 10, the introduction of the saturated Ca(OH)_2_ medium fundamentally altered the stabilising behaviour of SDBS observed in DI water. The GO/SDBS dispersion in Ca(OH)_2_ retained less than half of its initial absorbance at t = 60 min. This represents a large reduction compared to the equivalent dispersion in DI water, which retained above 90%, and the retention was similar to that of GO in saturated Ca(OH)_2_ without any surfactant. This indicates that SDBS provided no meaningful stabilisation under these conditions.

The absorbance declined continuously throughout the observation period, with a moderate decline in the first 20 min followed by a sharper decline between t = 20 and t = 60 min. This behaviour closely mirrored the destabilisation mechanism observed for GO in saturated Ca(OH)_2_ alone (Section 3.3). It suggests that the presence of SDBS did not effectively suppress Ca^2+^-mediated aggregation. This is consistent with the previous study [39], which showed that Ca^2+^ complexation with oxygen-containing functional groups of GO is the primary factor responsible for GO aggregation in an alkaline environment.

Visual inspection of the vials at 0, 30, and 60 min is shown in Figure 10 and supports the UV-Vis data. While the dispersion appeared homogeneous immediately after sonication, visible sedimentation was evident by t = 30 min, with substantial GO accumulation at the base of the vial at t = 60 min. This closely resembles the sedimentation behaviour observed for GO in saturated Ca(OH)_2_ without the surfactant.

#### 3.5.2. GO Dispersions with CTAB in Saturated Ca(OH)_2_

**Figure 11:**
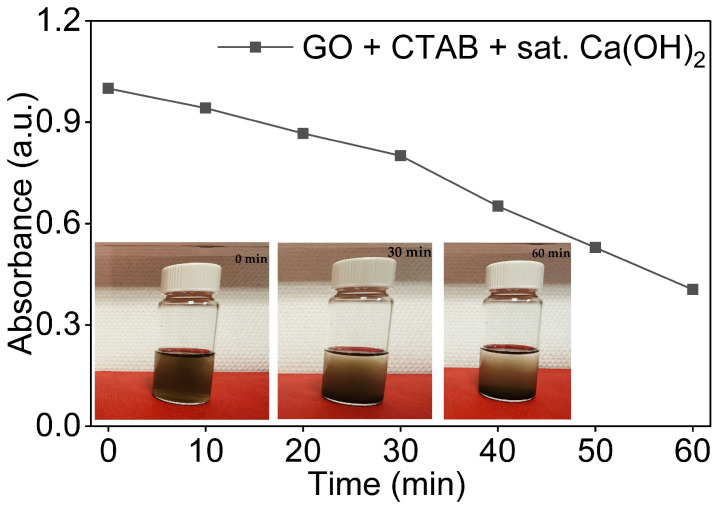
Normalised UV-Vis absorbance of 0.08 mg/mL GO dispersions in saturated Ca(OH)_2_ and CTAB as a function of time with visual observation at 0 min, 30 min, and 60 min.

The dispersion was prepared at a GO:CTAB ratio of 1:1 following the same sonication protocol as described earlier. As shown in Figure 11, the GO/CTAB dispersion in saturated Ca(OH)_2_ retained less than half of its initial absorbance at t = 60 min. This retention was similar to that of GO in Ca(OH)_2_ without the surfactant and to the GO/SDBS dispersion in the same medium, indicating that CTAB provided no meaningful stabilisation under these conditions.

The absorbance declined continuously throughout the observation period, with a moderate decline in the first 20 min followed by a sharper decline between t = 20 and t = 60 min. This behaviour confirmed that, even in the presence of CTAB, Ca^2+^-induced aggregation still occurred. As established in Section 3.4.2, CTAB partially neutralises the native surface charge of GO even in DI water. In the Ca^2+^-rich alkaline environment, this reduction in surface is further enhanced by Ca^2+^-driven double-layer compression and bridging flocculation between GO sheets [39], resulting in stability comparable to that of GO in Ca(OH)_2_ without additives.

Visual inspection of vials taken at 0, 30, and 60 min in Figure 11 showed a sedimentation pattern closely resembling that observed for GO in Ca(OH)_2_ alone, with clear sediment accumulation visible at t = 60 min, consistent with UV-Vis data.

### 3.6. Zeta Potential and Particle Size of GO Dispersions

**Figure 12:**
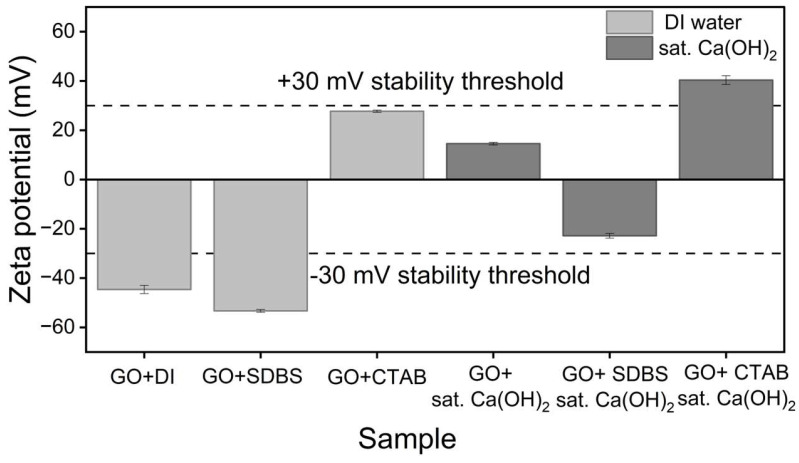
Zeta potential of GO dispersions in DI water and sat. Ca(OH)_2_, with and without ionic surfactants.

**Figure 13:**
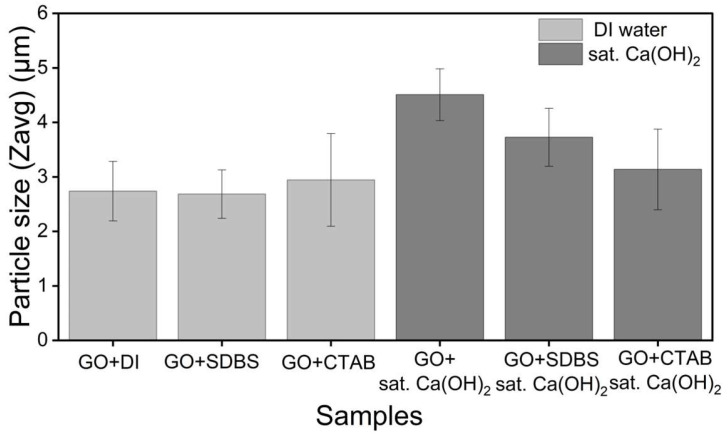
Particle size of GO dispersions in DI water and sat. Ca(OH)_2_, with and without ionic surfactants.

To gain further insight into the surface charge state and aggregate size of the GO dispersion systems, zeta potential and hydrodynamic particle size measurements were carried out for all six conditions. The pH of each suspension was also recorded to confirm the chemical environment in which the measurement was performed. The results are summarised in Table 1 and presented in Figure 12 and Figure 13. These data extend the UV-Vis sedimentation observations of Section 3.2, Section 3.3, Section 3.4 and Section 3.5 by providing direct information on the electrostatic state of GO and the size of the suspended fraction at the time of measurement.

#### 3.6.1. Zeta Potential and Particle Size in DI Water

In DI water, the three GO dispersion systems exhibited distinct zeta potential values consistent with their respective surfactant chemistries. Unsupplemented GO exhibited a strongly negative zeta potential of −44.6 ± 1.7 mV, attributable to the deprotonated carboxyl and hydroxyl groups on the GO surface. The addition of SDBS shifted the zeta potential to a more negative value of −53.3 ± 0.5 mV, indicating that the anionic sulphonate head groups of SDBS adsorbed onto the GO surface and reinforced the native negative surface charge.

The addition of CTAB produced a charge inversion, with the zeta potential shifting to a positive value of 25.7 ± 0.4 mV. This indicates that the cationic trimethylammonium head groups of CTAB adsorbed onto the negatively charged GO surface in sufficient quantity to overcompensate the native negative charge and impart a net positive charge to the GO sheets [60]. In all three systems, the zeta potential magnitude was sufficient to support the stable or near-stable dispersion observed by UV-Vis (Section 3.2 and Section 3.4).

The hydrodynamic particle sizes of the three water-based systems were all in the micrometer range, with Z-average values of 2.7 ± 0.5 μm, 2.6 ± 0.4 μm, and 2.9 ± 0.8 μm for GO/DI, GO/SDBS, and GO/CTAB, respectively. The polydispersity index (PDI) values were uniformly high (above 0.9 for all three systems), reflecting the broad size distribution typical of GO suspensions, in which sheets of varying lateral dimensions and degree of exfoliation coexist. As DLS assumes spherical particles, the Z-average values reported here represent an effective hydrodynamic diameter rather than a true physical dimension of GO sheets, but they remain useful as a comparative measure of the suspended fraction across the three systems. Together with the zeta potential results, these findings establish a baseline for surfactant behaviour in a low-ionic-strength environment and provide the reference point against which the dispersion behaviour in saturated Ca(OH)_2_, examined in Section 3.6.2, can be interpreted.

#### 3.6.2. Zeta Potential and Particle Size in Saturated Ca(OH)_2_

The zeta potential values measured in saturated Ca(OH)_2_ differed substantially from those in DI water. Unsupplemented GO in saturated Ca(OH)_2_ exhibited a positive zeta potential of 14.5 ± 0.5 mV, in contrast to the strongly negative value observed in DI water. This sign reversal indicates that Ca^2+^ adsorbed onto the GO surface in sufficient quantity to compensate for the native negative surface charge and produce a net positive value. The addition of SDBS in saturated Ca(OH)_2_ produced a zeta potential of −22.8 ± 1.0 mV, less negative than DI water but still indicating that some SDBS contribution to the surface charge was retained. The GO/CTAB system in Ca(OH)_2_ showed a zeta potential of 40.3 ± 1.8 mV, greater in magnitude than in DI water, consistent with a combined contribution from CTAB adsorption and Ca^2+^ binding to the GO surface.

The hydrodynamic particle sizes of the three Ca(OH)_2_ systems were larger than the corresponding values in DI water, consistent with the aggregation observed by UV-vis (Section 3.3 and Section 3.5). Unsupplemented GO in saturated Ca(OH)_2_ produced a Z-average of 4.5 ± 0.4 μm, the largest value among the six systems studied. The surfactant systems produced comparatively smaller Z-average values of 3.726 ± 0.5 μm for GO/SDBS/sat. Ca(OH)_2_ and 3.1 ± 0.7 μm for GO/CTAB/sat. Ca(OH)_2_, although all remained in the micrometer range. PDI values for the Ca(OH)_2_ systems were broad in all cases. The reduction in Z-average with surfactant addition suggests that the surfactants limited aggregation growth to some extent; however, neither prevented aggregation entirely.

## 4. Discussion

The dispersion of GO in DI water provided a stable baseline against which the effects of surfactants and Ca^2+^-rich alkaline environments could be evaluated. Both SDBS and CTAB at a mass ratio of 1:1 improved GO stability in DI water, with both systems retaining above 90% of their initial absorbance at 60 min. The shape of the decay profile, however, differed. GO with SDBS in DI water reached a stable plateau shortly after sonication, while GO with CTAB in DI water declined gradually throughout the observation period. The zeta potential results explain this difference. SDBS is an anionic surfactant; its hydrophobic tail adsorbs onto the GO surface, while its negatively charged sulphonate head points into the water [42,58]. This adds extra negative charge to the GO surface and produces a more negative zeta potential than unsupplemented GO. CTAB is a cationic surfactant. Its positively charged head adsorbs onto the negative GO surface and inverts the zeta potential to a positive value [43].

In saturated Ca(OH)_2_, the dispersion behaviour of GO was fundamentally different. The UV-Vis results showed that neither SDBS nor CTAB restored the stability observed in DI water. All three saturated Ca(OH)_2_ systems retained less than half of their initial absorbance at 60 min. The corresponding particle size data confirmed aggregation in all three systems. The zeta potential measurements alone, however, do not predict this behaviour. Unsupplemented GO in saturated Ca(OH)_2_ exhibited a small positive zeta potential, a sign reversal from its strongly negative value in DI water. This indicates that Ca^2+^ is adsorbed onto the GO surface in quantities sufficient to compensate for the native negative charge [61]. The GO with SDBS and GO with CTAB systems in saturated Ca(OH)_2_ retained zeta potentials of −22.8 mV and 40.3 mV, respectively. These magnitudes in conventional electrostatic terms would be expected to support some degree of colloidal stability. The UV-Vis results, however, showed that all three systems aggregated regardless. This indicates that the colloidal behaviour of GO in saturated Ca(OH)_2_ relevant to the cement system is not governed by net surface charge alone.

The aggregation observed in saturated Ca(OH)_2_ is consistent with chemical cross-linking of GO sheets through coordination between Ca^2+^ and carboxylate groups at the GO edges [61]. This mechanism has been identified as the primary cause of GO aggregation in cement pore solutions. This is further enhanced by the deprotonation of carboxyl groups at the high pH of saturated Ca(OH)_2_, which increases the number of -COO^−^ sites available for Ca^2+^ bridging [39]. Because this chemical bridging is a specific ion-functional group rather than an electrostatic one, ionic surfactants cannot suppress it, regardless of the charge type. The particle size data support this interpretation. The surfactant-containing systems showed somewhat smaller aggregates than unsupplemented GO in saturated Ca(OH)_2_, suggesting that the surfactants reduced aggregate size but did not prevent aggregation.

Previous studies have established Ca^2+^-GO complexation as a dominant aggregation mechanism in cement pore solutions [39]. The present study extends this picture by showing that the limited effectiveness of ionic surfactants in saturated Ca(OH)_2_ reflects the specific chemical nature of the Ca^2+^ -COO^−^ interaction rather than insufficient surface charge.

## 5. Conclusions

The following conclusions are drawn from this study:

- GO formed stable aqueous dispersions following probe ultrasonication at concentrations of 0.04, 0.06, and 0.08 mg/mL, with all three showing a two-phase profile of rapid initial settling followed by a stable plateau. The 0.08 mg/mL concentration was selected for further investigation.
- Saturated Ca(OH)_2_ markedly reduced GO colloidal stability, with 60 min absorbance retention decreasing from close to 70% in DI water to approximately 40% in saturated Ca(OH)_2_. The zeta potential of unsupplemented GO underwent a sign reversal from strongly negative in DI water to a small positive value in saturated Ca(OH)_2_, indicating substantial Ca^2+^ adsorption on the GO surface.
- Both SDBS and CTAB enhanced GO stability in DI water at a 1:1 GO:surfactant mass ratio, with both dispersions retaining above 90% of their initial absorbance at 60 min. The two surfactants modified the GO surface charge in opposite ways: SDBS reinforced the native negative charge to produce a more negative zeta potential, while CTAB inverted the surface charge to a positive value.
- Neither SDBS nor CTAB maintained GO colloidal stability in saturated Ca(OH)_2_. Despite producing zeta potentials of moderate to strongly positive magnitude, all three Ca(OH)_2_ systems retained less than half of their initial absorbance at 60 min. This indicates that aggregation in this environment is not governed by net surface charge alone.
- The combined UV-Vis, zeta potential, and particle size data indicate that the limited effectiveness of ionic surfactants in saturated Ca(OH)_2_ cannot be explained by the net surface charge alone and is consistent with the established mechanisms of Ca^2+^ chemical cross-linking of GO carboxyl groups. This is the central mechanistic finding of the present study and indicates that effective dispersants for GO in cement-relevant media must combine electrostatic and steric stabilisation. Future work will extend the present study to polymer-based dispersants and to the incorporation of stabilised GO into cement systems.

## Figures and Tables

**Table 1:** Zeta potential, particle size, and polydispersity index (PDI) of GO dispersions under different chemical environments.

Sample	pH	Size (Zavg) (μm)	Zeta Potential (mV)	PDI
GO + DI	4	2.7 ± 0.5	−44.6 ± 1.7	0.92 ± 0.12
GO + SDBS	4	2.6 ± 0.4	−53.3 ± 0.5	0.94 ± 0.03
GO + CTAB	3.9	2.9 ± 0.8	25.7 ± 0.4	0.96 ± 0.06
GO + sat. Ca(OH)_2_	12	4.5 ± 0.4	14.5 ± 0.5	0.35 ± 0.19
GO + SDBS + sat. Ca(OH)_2_	11.6	3.7 ± 0.5	−22.8 ± 1.0	0.84 ± 0.26
GO + CTAB + sat. Ca(OH)_2_	11.5	3.1 ± 0.7	40.3 ± 1.8	0.82 ± 0.11

## Data Availability

The original contributions presented in this study are included in the article/Supplementary Materials. Further inquiries can be directed to the corresponding author.

## Supplementary Materials

The following supporting information can be downloaded at https://www.mdpi.com/article/10.3390/nano16100632/s1. Figure S1: Full SEM micrograph of synthesised graphene oxide (GO); Figure S2: (Baseline Dispersion) Full UV-Vis absorption spectrum of (0.08 mg/mL) in DI water immediately after sonication; Figure S3: Full UV-Vis absorption spectrum of (0.08 mg/mL) in saturated Ca(OH)_2_ immediately after sonication; Table S1: Raw (non-normalised) UV–Vis absorbance values at 230 nm for GO dispersions in DI water at concentrations of 0.04, 0.06, and 0.08 mg/mL as a function of time following ultrasonication; Table S2: Raw (non-normalised) UV–Vis absorbance values at 230 nm for GO (0.08 mg/mL) in saturated Ca(OH)_2_, and with ionic surfactant additives (SDBS and CTAB at 1:1 GO:surfactant mass ratio) in both DI water and saturated Ca(OH)_2_, as a function of time following ultrasonication.

## Abbreviations

The following abbreviations are used in this manuscript:

GO	Graphene Oxide;
CNTs/CNFs	Carbon nanotubes/carbon nano-fibres;
SDBS	Sodium dodecylbenzene sulfonate;
CTAB	Cetyltrimethylammonium bromide;
UV-Vis	Ultraviolet-visible spectroscopy;
SEM	Scanning electron microscopy;
XRD	X-ray diffraction;
ATR-FTIR	Attenuated total reflectance Fourier transform infrared spectroscopy;
DI water	Deionised water;
Ca(OH)_2_	Calcium hydroxide;
C-S-H	Calcium silicate hydrate;
Ca^2+^	Calcium ion;
NaNO_3_	Sodium nitrate;
KMnO_4_	Potassium permanganate;
HCL	Hydrochloric acid;
H_2_O_2_	Hydrogen peroxide;
H_2_SO_4_	Hydrochloric acid;
a.u.	Arbitrary units;
-COO^−^	Carboxylate group/ion.

## References

[1] Neville A.M. (2011). Properties of Concrete.

[2] Hulagabali M.M., Vesmawala G.R., Patil Y.D. (2023). Synthesis, Characterization, and Application of Graphene Oxide and Reduced Graphene Oxide and Its Influence on Rheology, Microstructure, and Mechanical Strength of Cement Paste. J. Build. Eng..

[3] Alateah A.H. (2023). Graphene Concrete: Recent Advances in Production Methods, Performance Properties, Environmental Impact and Economic Viability. Case Stud. Constr. Mater..

[4] Pei C., Kong S., Guo M.-Z., Zhu J.-H. (2024). Water-Based Graphene Nanofluid Additives: Advancements in Sustainable, Low-Carbon, and High-Performance Nanocarbon-Modified Cementitious Materials. Cem. Concr. Res..

[5] Gholampour A., Kiamahalleh M.V., Tran D.N.H., Ozbakkaloglu T., Losic D. (2017). Revealing the Dependence of the Physiochemical and Mechanical Properties of Cement Composites on Graphene Oxide Concentration. RSC Adv..

[6] Chintalapudi K., Pannem R.M.R. (2020). The Effects of Graphene Oxide Addition on Hydration Process, Crystal Shapes, and Microstructural Transformation of Ordinary Portland Cement. J. Build. Eng..

[7] Sanchez F., Sobolev K. (2010). Nanotechnology in Concrete—A Review. Constr. Build. Mater..

[8] Sobolev K., Gutierrez M.F. (2005). How Nanotechnology Can Change the Concrete World. Am. Ceram. Soc. Bull..

[9] Li H., Xiao H.G., Yuan J., Ou J. (2004). Microstructure of Cement Mortar with Nano-Particles. Compos. B Eng..

[10] Miao X., Xing Y., Zheng H., Liu Q., Hu M., Guo J. (2023). Effects of Hybrid Graphene Oxide-Nanosilica on Calcium Silicate Hydrate in the Simulation Environment and Cement. ACS Omega.

[11] Tsardaka E.-C., Tsampali E., Stefanidou M. (2024). The Contribution of Nano-Alumina to Ultra-High-Performance Cement-Based Systems. Materials.

[12] Szleifer I., Yerushalmi-Rozen R. (2005). Polymers and Carbon Nanotubes—Dimensionality, Interactions and Nanotechnology. Polymer.

[13] Tyson B.M., Al-Rub R.K.A., Yazdanbakhsh A., Grasley Z. (2011). Carbon Nanotubes and Carbon Nanofibers for Enhancing the Mechanical Properties of Nanocomposite Cementitious Materials. J. Mater. Civ. Eng..

[14] Qiu L., Yang X., Gou X., Yang W., Ma Z.F., Wallace G.G., Li D. (2010). Dispersing Carbon Nanotubes with Graphene Oxide in Water and Synergistic Effects between Graphene Derivatives. Chem. Eur. J..

[15] Geim A.K., Novoselov K.S. (2007). The Rise of Graphene. Nat. Mater..

[16] Schulte J., Jiang Z., Sevim O., Ozbulut O.E. (2022). Graphene-Reinforced Cement Composites for Smart Infrastructure Systems.

[17] Zheng Q., Han B., Cui X., Yu X., Ou J. (2017). Graphene-Engineered Cementitious Composites: Small Makes a Big Impact. Nanomater. Nanotechnol..

[18] Dimov D., Amit I., Gorrie O., Barnes M.D., Townsend N.J., Neves A.I.S., Withers F., Russo S., Craciun M.F. (2018). Ultrahigh Performance Nanoengineered Graphene–Concrete Composites for Multifunctional Applications. Adv. Funct. Mater..

[19] Meng S., Ouyang X., Fu J., Niu Y., Ma Y. (2021). The Role of Graphene/Graphene Oxide in Cement Hydration. Nanotechnol. Rev..

[20] Salami B.A., Mukhtar F., Ganiyu S.A., Adekunle S., Saleh T.A. (2023). Graphene-Based Concrete: Synthesis Strategies and Reinforcement Mechanisms in Graphene-Based Cementitious Composites (Part 1). Constr. Build. Mater..

[21] Krystek M., Pakulski D., Patroniak V., Górski M., Szojda L., Ciesielski A., Samorì P., Krystek M., Górski M., Szojda L. (2019). High-Performance Graphene-Based Cementitious Composites. Adv. Sci..

[22] Krystek M., Ciesielski A., Samorì P. (2021). Graphene-Based Cementitious Composites: Toward Next-Generation Construction Technologies. Adv. Funct. Mater..

[23] Shamsaei E., de Souza F.B., Yao X., Benhelal E., Akbari A., Duan W. (2018). Graphene-Based Nanosheets for Stronger and More Durable Concrete: A Review. Constr. Build. Mater..

[24] Jing G., Wu J., Lei T., Wang S., Strokova V., Nelyubova V., Wang M., Ye Z. (2020). From Graphene Oxide to Reduced Graphene Oxide: Enhanced Hydration and Compressive Strength of Cement Composites. Constr. Build. Mater..

[25] Lin Y., Du H. (2020). Graphene Reinforced Cement Composites: A Review. Constr. Build. Mater..

[26] Pan Z., He L., Qiu L., Korayem A.H., Li G., Zhu J.W., Collins F., Li D., Duan W.H., Wang M.C. (2015). Mechanical Properties and Microstructure of a Graphene Oxide–Cement Composite. Cem. Concr. Compos..

[27] Suo Y., Guo R., Xia H., Yang Y., Zhou B., Zhao Z. (2022). A Review of Graphene Oxide/Cement Composites: Performance, Functionality, Mechanisms, and Prospects. J. Build. Eng..

[28] Suo Y., Guo R., Xia H., Yang Y., Yan F., Ma Q. (2020). Study on Modification Mechanism of Workability and Mechanical Properties for Graphene Oxide-Reinforced Cement Composite. Nanomater. Nanotechnol..

[29] Fonseka I., Mohotti D., Wijesooriya K., Lee C.K., Mendis P. (2024). Influence of Graphene Oxide Properties, Superplasticiser Type, and Dispersion Technique on Mechanical Performance of Graphene Oxide-Added Concrete. Constr. Build. Mater..

[30] Chen X., Qu Z., Liu Z., Ren G. (2022). Mechanism of Oxidization of Graphite to Graphene Oxide by the Hummers Method. ACS Omega.

[31] Zhao L., Guo X., Song L., Song Y., Dai G., Liu J. (2020). An Intensive Review on the Role of Graphene Oxide in Cement-Based Materials. Constr. Build. Mater..

[32] Lv S., Ma Y., Qiu C., Zhou Q. (2013). Regulation of Go on Cement Hydration Crystals and Its Toughening Effect. Mag. Concr. Res..

[33] Lv S., Ma Y., Qiu C., Sun T., Liu J., Zhou Q. (2013). Effect of Graphene Oxide Nanosheets of Microstructure and Mechanical Properties of Cement Composites. Constr. Build. Mater..

[34] Liao C., Lin B., Li M., Dai G., Hu S. (2024). Synergistic Effects of Graphene Oxide and Fly Ash on Rheology, Mechanical Properties, and Microstructure of Highly-Flowable Cementitious Grouts. J. Build. Eng..

[35] Chen X., Park J., Ji W., Huang Y., Lu J.X., Hu Z., Poon C.S. (2025). Unlocking the Interaction Mechanism of CNTs and C-S-H on Enhancing Elastic and Viscoelastic Properties of Alite Paste. Adv. Sci..

[36] Wei X.X., Pei C., Zhu J.H. (2024). Towards the Large-Scale Application of Graphene-Modified Cement-Based Composites: A Comprehensive Review. Constr. Build. Mater..

[37] Zhao L., Guo X., Liu Y., Ge C., Chen Z., Guo L., Shu X., Liu J. (2018). Investigation of Dispersion Behavior of GO Modified by Different Water Reducing Agents in Cement Pore Solution. Carbon.

[38] Liu C., Huang X., Wu Y.Y., Deng X., Zheng Z., Xu Z., Hui D. (2021). Advance on the Dispersion Treatment of Graphene Oxide and the Graphene Oxide Modified Cement-Based Materials. Nanotechnol. Rev..

[39] Zhao L., Zhu S., Wu H., Zhang X., Tao Q., Song L., Song Y., Guo X. (2020). Deep Research about the Mechanisms of Graphene Oxide (GO) Aggregation in Alkaline Cement Pore Solution. Constr. Build. Mater..

[40] Liu J., Fu J., Yang Y., Gu C. (2019). Study on Dispersion, Mechanical and Microstructure Properties of Cement Paste Incorporating Graphene Sheets. Constr. Build. Mater..

[41] Lotya M., King P.J., Khan U., De S., Coleman J.N. (2010). High-Concentration, Surfactant-Stabilized Graphene Dispersions. ACS Nano.

[42] Fernández-Merino M.J., Paredes J.I., Villar-Rodil S., Guardia L., Solís-Fernández P., Salinas-Torres D., Cazorla-Amorós D., Morallón E., Martínez-Alonso A., Tascón J.M.D. (2012). Investigating the Influence of Surfactants on the Stabilization of Aqueous Reduced Graphene Oxide Dispersions and the Characteristics of Their Composite Films. Carbon.

[43] Meng W., Gall E., Ke F., Zeng Z., Kopchick B., Timsina R., Qiu X. (2015). Structure and Interaction of Graphene Oxide- Cetyltrimethylammonium Bromide Complexation. J. Phys. Chem. C.

[44] Zhang F., Li S., Zhang Q., Liu J., Zeng S., Liu M., Sun D. (2019). Adsorption of Different Types of Surfactants on Graphene Oxide. J. Mol. Liq..

[45] Chuah S., Li W., Chen S.J., Sanjayan J.G., Duan W.H. (2018). Investigation on Dispersion of Graphene Oxide in Cement Composite Using Different Surfactant Treatments. Constr. Build. Mater..

[46] Jing G.J., Ye Z.M., Lu X.L., Wu J.M., Wang S.X., Cheng X. (2018). Incorporating Graphene Oxide into Lime Solution: A Study of Flocculation and Corresponding Improvement. Mater. Construcc..

[47] Hummers W.S., Offeman R.E. (1958). Preparation of Graphitic Oxide. J. Am. Chem. Soc..

[48] Zhou Y., Bao Q., Tang L.A.L., Zhong Y., Loh K.P. (2009). Hydrothermal Dehydration for the “Green” Reduction of Exfoliated Graphene Oxide to Graphene and Demonstration of Tunable Optical Limiting Properties. Chem. Mater..

[49] Ferrari A.C., Robertson J. (2000). Interpretation of Raman Spectra of Disordered and Amorphous Carbon. Phys. Rev. B.

[50] Zhu Y., Murali S., Cai W., Li X., Suk J.W., Potts J.R., Ruoff R.S. (2010). Graphene and Graphene Oxide: Synthesis, Properties, and Applications. Adv. Mater..

[51] Zhang Z., Schniepp H.C., Adamson D.H. (2019). Characterization of Graphene Oxide: Variations in Reported Approaches. Carbon.

[52] Shahriary L., Athawale A.A. (2014). Graphene Oxide Synthesized by Using Modified Hummers Approach. Int. J. Renew. Energy Environ. Eng..

[53] Konios D., Stylianakis M.M., Stratakis E., Kymakis E. (2014). Dispersion Behaviour of Graphene Oxide and Reduced Graphene Oxide. J. Colloid Interface Sci..

[54] Ayán-Varela M., Paredes J.I., Guardia L., Villar-Rodil S., Munuera J.M., Díaz-González M., Fernández-Sánchez C., Martínez-Alonso A., Tascón J.M.D. (2015). Achieving Extremely Concentrated Aqueous Dispersions of Graphene Flakes and Catalytically Efficient Graphene-Metal Nanoparticle Hybrids with Flavin Mononucleotide as a High-Performance Stabilizer. ACS Appl. Mater. Interfaces.

[55] Lothenbach B., Winnefeld F. (2006). Thermodynamic Modelling of the Hydration of Portland Cement. Cem. Concr. Res..

[56] Dreyer D.R., Park S., Bielawski C.W., Ruoff R.S. (2010). The Chemistry of Graphene Oxide. Chem. Soc. Rev..

[57] Mohammed A., Sanjayan J.G., Duan W.H., Nazari A. (2015). Incorporating Graphene Oxide in Cement Composites: A Study of Transport Properties. Constr. Build. Mater..

[58] Lotya M., Hernandez Y., King P.J., Smith R.J., Nicolosi V., Karlsson L.S., Blighe F.M., De S., Wang Z., McGovern I.T. (2009). Liquid Phase Production of Graphene by Exfoliation of Graphite in Surfactant/Water Solutions. J. Am. Chem. Soc..

[59] Konkena B., Vasudevan S. (2012). Understanding Aqueous Dispersibility of Graphene Oxide and Reduced Graphene Oxide through p K a Measurements. J. Phys. Chem. Lett..

[60] Chen H., Wang D., Wang X., Ye Z., Han L., Xu Q. (2020). Triple Phase Inversion of Emulsions Stabilized by Amphiphilic Graphene Oxide and Cationic Surfactants. ACS Omega.

[61] Park S., Lee K.S., Bozoklu G., Cai W., Nguyen S.B.T., Ruoff R.S. (2008). Graphene Oxide Papers Modified by Divalent Ions—Enhancing Mechanical Properties via Chemical Cross-Linking. ACS Nano.

