# Supplementary material for: Dispersion of Graphene Oxide: Evaluating Ionic Surfactants for Nanocomposite Cement Applications
Source: Nanomaterials (Basel). 2026 May 19;16(10):632. doi: 10.3390/nano16100632 (PMC13209221; doi:10.3390/nano16100632)
Supplement: Supplementary file 1 [file nanomaterials-16-00632-s001.zip › nanomaterials-4259766-supplementary.pdf]

Supplementary Materials:

Dispersion of Graphene Oxide: Evaluating Ionic Surfactants for Nanocomposite Cement Applications

Supplementary Figures

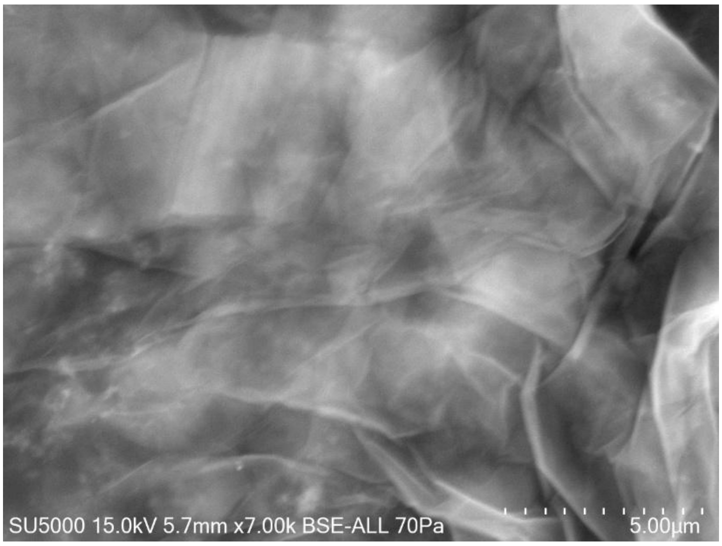

Figure S1. Full SEM micrograph of synthesised graphene oxide (GO).

(Baseline dispersion) Full UV-Vis absorption spectrum of GO (0.08 mg/mL) in DI water immediately after sonication

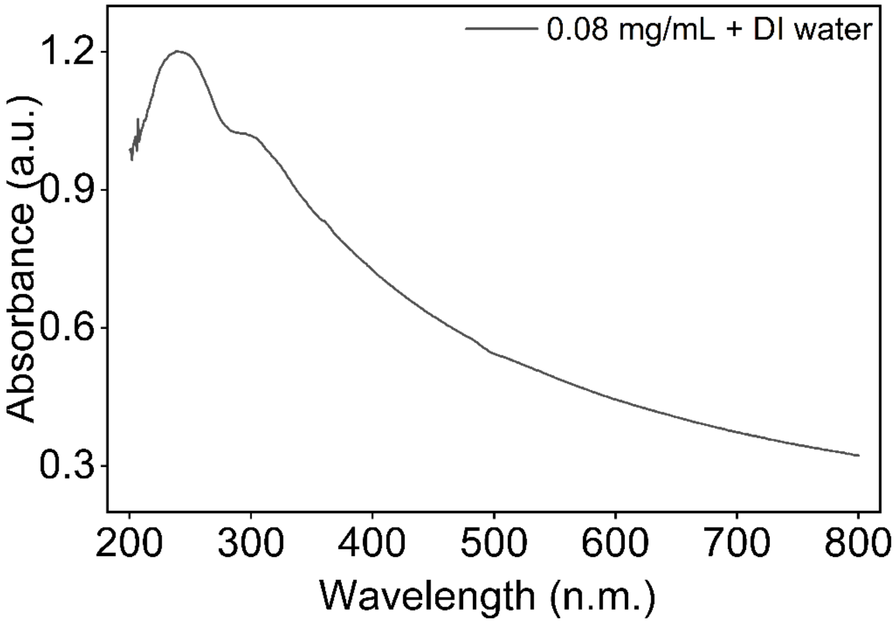

Figure S2. Full UV–Vis absorption spectrum of GO at 0.08 mg/mL in DI water immediately after sonication. The spectrum shows the characteristic π–π* transition at 230 nm and the n–π* transition shoulder at approximately 300 nm.

Full UV-Vis absorption spectrum of GO (0.08 mg/mL) in saturated $Ca(OH)_2$ immediately after sonication

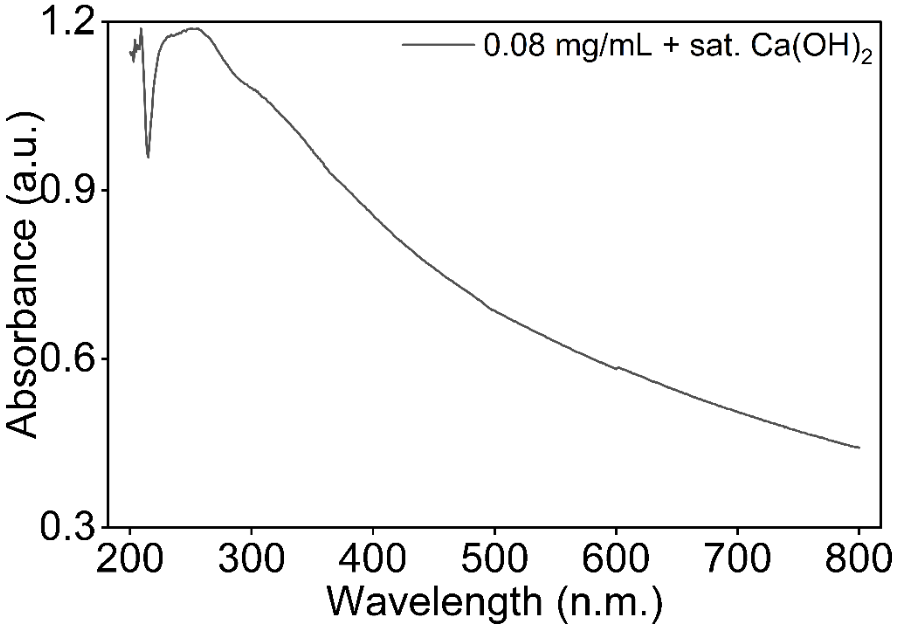

Figure S3. Full UV–Vis absorption spectrum of GO at 0.08 mg/mL in saturated $Ca(OH)_2$ immediately after sonication, demonstrating the retention of the characteristic GO absorption peak at 230 nm before aggregation.

Supplementary Tables

Table S1. Raw (non-normalised) UV–Vis absorbance values at 230 nm for GO dispersions in DI water at concentrations of 0.04, 0.06, and 0.08 mg/mL as a function of time following ultrasonication.

Time (min)	0.04 mg/mL	0.06 mg/mL	0.08 mg/mL
0	0.600	0.902	1.183
10	0.409	0.654	0.863
20	0.409	0.644	0.841
30	0.407	0.635	0.833
40	0.403	0.627	0.823
50	0.401	0.632	0.823
60	0.396	0.629	0.823

Table S2. Raw (non-normalised) UV–Vis absorbance values at 230 nm for GO (0.08 mg/mL) in saturated $Ca(OH)_2$, and with ionic surfactant additives (SDBS and CTAB at 1:1 GO: surfactant mass ratio) in both DI water and saturated $Ca(OH)_2$, as a function of time following ultrasonication.

Time (min)	GO/ $Ca(OH)_2$	GO + SDBS / DI water	GO + CTAB / DI water	GO + SDBS / $Ca(OH)_2$	GO + CTAB / $Ca(OH)_2$
0	1.172	1.347	1.143	0.926	1.136
10	1.123	1.330	1.134	0.867	1.070
20	1.098	1.346	1.100	0.817	0.985
30	0.943	1.310	1.092	0.707	0.910
40	0.786	1.289	1.074	0.680	0.740
50	0.604	1.300	1.051	0.577	0.601
60	0.492	1.274	1.047	0.425	0.460
